# Supplementary material for: Multitask feature learning approach for knowledge graph enhanced recommendations with RippleNet
Source: PLoS One. 2021 May 14;16(5):e0251162. doi: 10.1371/journal.pone.0251162 (PMC8121330; doi:10.1371/journal.pone.0251162)
Supplement: Attachment — Submitted filename: Response to Reviewers.doc [file pone.0251162.s001.doc]

**Original Manuscript ID:** PONE-D-21-07884

**Original Article Title: “**Multitask Feature Learning Approach for Knowledge Graph Enhanced Recommendations with RippleNet”

**To:** PLOS ONE Editor

**Re:** Response to reviewers

Dear Editor,

Thank you for allowing a resubmission of our manuscript, with an opportunity to address the reviewers’ comments.

We are uploading (a) our point-by-point response to the comments (below) (response to reviewers), (b) an updated manuscript with yellow highlighting indicating changes, and (c) a clean updated manuscript without highlights.

Best regards,

<YueQun Wang;Hao Zhang > et al.

**Reviewer#1, Concern # 1:** In Sec.2, the Related Work are introduced. While displaying the relevant technologies, it is also necessary to clarify why these technologies are used. For example, explain the role of Side information and so on.

**Author response:** Thank the reviewers for your comments. As for the design idea and concept of the model proposed in this paper, there is not much elaboration in this paper's second chapter. We will give a reasonable explanation in the first section of the second chapter of this paper.

**Author action:** We updated the manuscript by giving the reasons and technical basis for combining the model and knowledge graph as side information in Sec.2.

**Reviewer#1, Concern # 2:** As for the illustration of the proposed model, fig2 is not a good one. Firstly, it’s hard to recognize the input of the model and the relation between different modules. Secondly, the meanings of symbols and different color parts are not given.

**Author response:** Thank the reviewers for your comments. We are sorry that the model presented in Figure 2 is not explained in-depth in this paper.

**Author action:** We updated the manuscript by explaining Fig. 2, including the meaning of each color module and the fusion process of the knowledge graph and recommendation system.

**Reviewer#1, Concern # 3:** The same symbol expresses too much meaning, for example, the meaning of L and K in Eq.4 is not elaborated.

**Author response:** Thank you for your comments. This issue has been modified and the full text reviewed.

**Author action:** We updated the manuscript by revising the full text for this type of mistake.

**Reviewer#1, Concern # 4:** The figure presented in this paper needs to be explained as comprehensively as possible. Include the meaning of each module and the links between the modules, should be given as comprehensive as possible

**Author response:** This is a question which indeed deserves our attention. The interpretation of Figure 2 has been answered and modified in Concern 2, and the full text has been reviewed to give a reasonable explanation of each figure.

**Author action:** We updated the manuscript by adding an explanation of Figures 2 and 3.

**Reviewer#1, Concern # 5:** As we know, DKN is designed specially for news recommendation where the entities are extracted from news titles. Obviously less entities could be extracted from movie names since they are often shorter than news titles. These need to be explained in the results. The experiment shows that the accuracy of recommendation is greatly improved.

**Author response:** Thank you for your comments. DKN is a depth model designed for news recommendations. This paper introduces DKN into baseline because DKN is also a model that combines depth recommendation with a knowledge graph. Besides, the MKR model and RippleNet model are introduced as baseline. Although DKN is a depth model with limited application, it is also representative. We will explain and indicate the limitations of DKN in the experimental results section and the reasons for using DKN as baseline

**Author action:** We updated the manuscript by explaining the limitations of DKN and the reasons for using DKN as the baseline.

**Reviewer#2, Concern # 1:** There are many notations in this paper, which should be clearly defined: What do L and K represent respectively in Eq.4?What do o_u^1 represent respectively in Eq.11?

**Author response:** Thank you for your comments. We have revised the above issues and reviewed the full text to revise the same issues

**Author action:** We updated the manuscript by giving an explanation of all the symbols.

**Reviewer#2, Concern # 2:** Some misused words need to be corrected such as:Recommendation Modul ->Recommendation Module;KGE Modul ->KGE Module;“The relationship structure between the knowledge graph and the recommender system is shown in Tab.1” Should change Tab.1 to Fig.1.

**Author response:** Thank you for your comments. We have revised the above issues and reviewed the full text to revise the same issues.

**Author action:** We updated the manuscript by modifying and checking the errors mentioned above.

**Reviewer#2, Concern # 3:** Since Figure 2 is the main structure of the model proposed in this paper, it needs to be introduced emphatically. Figure 2 should clarify the inputs and outputs of the model, the meaning of the symbols and the parts with different colors need to be explained.

**Author response:** Thank the reviewers for your comments. We are sorry that the model presented in Figure 2 is not explained in-depth in this paper.

**Author action:** We updated the manuscript by explaining Fig. 2, including the meaning of each color module and the fusion process of the knowledge graph and recommendation system.

**Reviewer#2, Concern # 4:** The format of the Figure mentioned in the body should be uniform. Cite figures with the format: Fig 1A, Fig 1B, Fig 2, Fig 3, etc. The cite to Figure 2 and Figure 3 in this article is incorrectly formatted. The name of Figure 1 is too long, so there is no need to explain the figure in the title.

**Author response:** We are grateful to the reviewers for their careful review of my paper. The referencing of the figures has been consolidated and the title of each figures has been modified.

**Author action:** We updated the reference format by unifying the form and changed the title of Figure 1

**Reviewer#2, Concern # 5:** In Sec.6, the parameter of baseline should give reasonable meanings. Such as explain the meaning of λ in each baseline model.

**Author response:** Thank you for your comments. We have revised the above issues and reviewed the full text to revise the same issues.

**Author action:** We updated the manuscript by giving the parameter of baseline should give reasonable meanings in Sec.6.

**Reviewer#3, Concern # 1:** There is no need to introduce the details of recommender system (RS) categories, side information, etc. in Sec.1, which should be removed into Sec.2. For the Sec.1, only a few technologies need to be mentioned, mainly explaining the significance and purpose of the model design.

**Author response:** We are grateful to the reviewers for their careful review of my paper. We will revise it according to your suggestion.

**Author action:** We updated the manuscript by modifying the contents of Chapter 1 and Chapter 2 so that Chapter 2 highlights the technical issues and Chapter 1 shows the basic ideas of the model

**Reviewer#3, Concern # 2:** The authors should elaborate more about their solution's motivations and rationales. For example, why did they combine joint learning and alternating learning? What is the advantage of such process compared with previous KG-based RS? What is the objective of corss&compress units?

**Author response:** Thank the reviewers for their opinions. We will add a reasonable explanation to the model idea in the first chapter

**Author action:** We updated the manuscript by elaborating more about our solution's motivations and rationales in Sec .1.

**Reviewer#3, Concern # 3:** Notations and explanations should not only be shown in the table, but also explained in terms of the notation after the formula is listed. Although it has been mentioned above, the explanation of important formula symbols still needs to be mentioned again.

**Author response:** We are grateful to the reviewers for their careful review of my paper. This issue has been modified and the full text reviewed.

**Author action:** We updated the manuscript by revising the full text for this type of mistake.

**Reviewer#3, Concern # 4:** The writing of this paper is somewhat lacking due to some grammar and format errors and typos So I suggest the authors proof read their manuscript.

**Author response:** Thank you for your careful review of our article. For grammar problems, we will review the full text and correct grammatical errors.

**Author action:** We updated the manuscript by correcting any grammatical errors in the text.

**Reviewer#3, Concern # 5:** Figure 2~5 cannot be found in the paper, only figure title here.

**Author response:** Thank the reviewers for their opinions. During the submission process, the figure is submitted separately from the paper, the figure at the end of the Manuscript.

**Author action:** We updated the manuscript by doing nothing.
